# Shifts in rotifer life history in response to stable isotope enrichment: testing theories of isotope effects on organismal growth

**DOI:** 10.1098/rsos.160810

**Published:** 2017-03-29

**Authors:** Elena Gorokhova

**Affiliations:** Department of Environmental Science and Analytical Chemistry, Stockholm University, Svante Arrhenius väg 8, 10691 Stockholm, Sweden

**Keywords:** stable isotopes, ^15^N-labelling, growth and life histories, longevity, isotope resonance theory, kinetic isotope effect

## Abstract

In ecology, stable isotope labelling is commonly used for tracing material transfer in trophic interactions, nutrient budgets and biogeochemical processes. The main assumption in this approach is that the enrichment with a heavy isotope has no effect on the organism growth and metabolism. This assumption is, however, challenged by theoretical considerations and experimental studies on kinetic isotope effects *in vivo*. Here, I demonstrate profound changes in life histories of the rotifer *Brachionus plicatilis* fed ^15^N-enriched algae (0.4–5.0 at%); i.e. at the enrichment levels commonly used in ecological studies. These findings support theoretically predicted effects of heavy isotope enrichment on growth, metabolism and ageing in biological systems and underline the importance of accounting for such effects when using stable isotope labelling in experimental studies.

## 1. Introduction

Stable isotopes are safe and non-radioactive, occurring naturally in the environment. They do not decay, which makes them attractive natural tracers in experimental studies [1,2]. Naturally occurring differences in isotopic signatures (*δ*-values) between the substrate and the product of the reaction are used in ecology and biogeochemistry to understand flows and processes. Another approach is enrichment studies that deploy compounds enriched in particular isotopes to the system and follow their fate. Such stable isotope labelling is commonly used for tracing material transfer in trophic interactions, material flows and biogeochemical processes. It may also indicate whether systems in question can metabolize certain compounds. There is a variety of commercially available compounds with the heavy isotope making up 99% of the tracer element. These compounds can be integrated into a nutrient medium for primary producers and feed regimes of consumers when addressing, for example, nutrient uptake, prey selectivity and fate of dietary nutrients [2]. One should keep in mind, however, that natural abundance and enrichment studies are often subject to different assumptions and caveats, and many of them are not sufficiently understood.

In biota, heavy isotopes of common elements (C, H, N and O) exist in much lower abundance than their light counterparts (1.1% for ^13^C, 0.015% for ^2^H or D, 0.2% for ^18^O and 0.37% for ^15^N). Despite their small contribution, heavy isotopes produce remarkable kinetic difference because the gain in weight to charge increases the stability of heavy isotope-containing chemical bonds, which may substantially slow their reactivity—this phenomenon is called the kinetic isotope effect (KIE). As light isotopes engage more easily in chemical reactions, the relative abundance of heavy stable isotopes increases in the substrate, leading to progressively slower reactions [3]. However, one of the main assumptions of the isotope-enrichment approach is that the increased levels of a heavy isotope have no effect on the organism growth and metabolism. This assumption is challenged by both theoretical and empirical research. In particular, alterations in growth and metabolic profiles induced by ^13^C- and/or ^15^N-enriched media were reported for bacteria [4–6], algae [7] and fungi [8]. A hypothesis has been put forward that the increased uptake of the heavy isotope in a biological system would affect the kinetics of biochemical reactions, increase the stability of the biomolecules and organism longevity [9,10]. Supporting this view, the relative abundance of heavy isotope-containing metabolites in yeast exhibited an ageing-associated trend, with most abundant amino acids declining in their ^13^C and ^2^H content in senescent cultures [11]. The authors hypothesized that cells might gradually lose the ability to retain heavy metabolites with ageing and, by introducing D_2_O in the culture medium, they were able to slow down this metabolic deterioration and ageing.

Further, the isotopic resonance theory advocates a non-monotonous dependence of the reaction rate upon the enrichment [12]. The proposed mechanism relates to the overall reduction of the system's complexity, represented by a total number of distinct quantum mechanical states in the macromolecules and analysed using a peptide mass distribution. This theory predicts that at certain abundances of the stable isotopes, the rates of chemical and biochemical reactions accelerate, affecting biological growth. For example, the resonance conditions are predicted to occur at 0.35% of ^13^C, 3.5% of ^15^N, 6.6% of ^18^O and 0.03% of ^2^H, and these predictions were confirmed using experiments with *E. coli* grown at varying isotopic composition [6]. Although these resonance isotopic concentrations are too high to occur naturally in any environment on Earth, they are within the range of concentrations applied in laboratory and field experiments with isotope-enriched substrates (e.g. [13–17]). For studies using isotope-enriched substrates, the occurrence of KIEs and resonance conditions imply that exposing producers or consumers to nutrient medium or food with isotopic signatures greatly deviating from their natural levels can cause alterations in organism growth and metabolism. If present, these alterations may affect the experimental outcome and lead to flawed conclusions. Therefore, understanding KIEs on growth and metabolism across different levels of biological organization would facilitate application of stable isotope approach in ecological and metabolomic studies.

Here, we used parthenogenic rotifer *Brachionus plicatilis* grown on ^15^N-enriched algae and assayed feeding, fecundity, lifespan and quality of the offspring at the ^15^N concentration range of 0.37 at% (natural abundance; δ^15^N = −2.7‰) to 5 at% (heavily enriched; δ^15^N = 13 172‰). The study provides the first empirical evidence that the chronic intake of ^15^N-enriched food can cause various changes in animal life history, including beneficial effects on lifespan and detrimental effects on reproduction. Moreover, at theoretically predicted resonance (3.5 at% ^15^N), the reproduction and offspring quality were elevated in comparison with other enriched diets. Our findings support the existence of an array of growth-related responses to ^15^N concentration in the diet and highlight the importance of accounting for these effects when designing experimental studies with ^15^N-labelling.

## 2. Material and methods

### 2.1. Test organisms

Rotifers are particularly well suited for testing life-history traits because of their short lifespan, parthenogenic reproduction and ease of culture [18]. The rotifers *Brachionus plicatilis* Müller (*Nevada* strain, originally from SINTEF Center of Aquaculture Norway) were cultured in 1 l glass beakers at 15 ppt ASW (artificial seawater, Instant Ocean), constant illumination (30 µE cm^−2^ s^−1^) and temperature (26 ± 2°C). The animals were fed with marine microalga *Isochrysis galbana* Parke 1949 grown in F medium [19]; the food was provided daily at a density of 5 × 10^6^ cell ml^−1^. Under these conditions, the rotifers have a pre-reproductive (neonate) stage of 1–2 days, reach maximum reproductive output at day 4–5, produce 16–20 eggs and live 10–12 days (personal observation), which is in the range of variation for this species [20]. All animals used in the study reproduced asexually; mictic females or males were not observed.

### 2.2. Experimental diets

For the experimental diets, the algae were enriched with ^15^N by replacing Na^14^NO_3_ with Na^15^NO_3_ (98 at% pure, Aldrich, St. Louis, MO, USA) in the medium; 10% of NaNO_3_ was enriched with ^15^N. The algae were grown under standard light and temperature conditions with constant shaking, and the isotope-enriched solution was added 5 days before harvesting, to ensure sufficient uptake. This labelling procedure yielded a stock solution of algae with δ^15^N values of 16 190‰ (6 at% ^15^N). The δ^15^N values as well as elemental concentrations in the labelled (C%: 40.2 ± 1.2% and N%: 6.2 ± 0.6%) and unlabelled (C%: 41.1 ± 1.3% and N%: 6.8 ± 0.5%) algae with three replicate samples per group were determined at the UC Davis Stable Isotope Facility, USA. There was no significant difference for either C% (*t*-test: *t*_4_ = 0.85; *p* > 0.45) or N% (*t*_4_ = 1.33; *p* > 0.25) between the labelled and unlabelled algae. Therefore, all experimental diets were assumed to be equally nutritious. To prepare the experimental diets, the labelled and unlabelled algae were mixed to correspond to 0.4, 0.5, 1, 3.5 and 5 at% of ^15^N; the unlabelled algae (δ^15^N: −2.7‰ corresponding to 0.37 at%) were used as a control. For each food batch, the algal concentrations were determined using a laser particle counter Spectrex PC-2000 (Spectrex Corp., CA, USA).

### 2.3. Life tables: experimental set-up and measured endpoints

For each experiment, resting eggs were hatched for 20–22 h in 20 ml ASW into a physiologically uniform cohort of neonates. The life table experiment was carried out in 24-well tissue culture microplates containing 2 ml culture medium, with one newly hatched female *B. plicatilis* per well (24 wells per treatment). The experiment consisted of six treatments corresponding to ^15^N in the experimental diets. The algae were provided at 2 × 10^6^ cell ml^−1^, and the maternal females were transferred to a new medium every second day. Plates were checked daily until the last animal died, mortality was recorded, and the offspring were counted and removed. Body growth in this species follows the Bertalanffy function, reaching 85–90% of maximum size between the first and second day of life [21]. Therefore, the neonate (130–170 µm) can easily be distinguished from the maternal female (200–250 µm) during the first 24 h after hatching. The offspring originated from the same female were pooled and preserved in RNA later for biochemical measurements. All plates were kept in the dark at 26°C in climate-control chambers.

### 2.4. Feeding rate

To examine whether the ^15^N-labelling affects individual food intake, a feeding experiment was conducted using 24-well microplates with 10 individuals per well, 18 replicate wells with rotifers and six control wells (algae only) per treatment. To prevent the hatching of amictic eggs and thus keep a constant number of individuals per well, rotifers were also treated with 20 µM 5-fluoro-2-deoxyuridine (FDU) [22]. The treatments were represented by the same diets as in the life table experiment, 1 ml per well, 5 × 10^5^ cell ml^−1^. This concentration represented average food concentration occurring in the life table experiment between the media renewals; these conditions were used to ensure saturating food levels, a constant number of rotifers per well, and, hence, constant intake rate during the incubation [23]. The plates were incubated in darkness for 6 h and the filtering rate was calculated from the cell densities measured with the particle counter before and after the incubation.

### 2.5. Biochemical assays

We measured RNA:protein ratio and individual protein content as indices of growth and condition in the offspring using the neonates collected during the life table experiment. The animals from each sample were divided between the two assays: 5–15 individuals were used in RiboGreen assay measuring RNA quantity [24] and 5–10 individuals in NanoOrange assay measuring protein quantity [25]. Using RiboGreen® Quantitation Kit (Invitrogen, Molecular Probes™), the RNA was quantified after extraction with N-laurylsarcosine followed by RNase digestion [26]. Fluorescence measurements were done in triplicate for each sample, standard, and negative control using FLUOstar Optima reader (BMG Labtech, Germany) with 485 nm for excitation and 520 nm for emission and black solid flat-bottom microplates (Greiner Bio-One GmbH). For protein measurements with NanoOrange® Protein Quantitation Kit (Invitrogen, Molecular Probes™), Bovine serum albumin (BSA, Molecular Probes) was used as a protein standard. The samples were analysed according to the manufacturer guidelines using the same type of microplates, plate reader and wavelengths as for the RNA quantification.

### 2.6. Statistics and data analysis

The life-history parameters used for treatment effect analysis were: age-specific fecundity, reproductive period (the duration between the first and last offspring production), lifetime fecundity (total number of offspring produced by a female) and lifespan calculated from the survival curve. The time of death was determined as the midpoint between the last observation before death and the first after that. Kaplan–Meier survival functions and daily reproduction curves were used to analyse the differences between the treatments using Mantel–Cox log-rank tests as implemented in Prism v. 6.0 (GraphPad). For other endpoints, D'Agostino & Pearson omnibus normality test was performed to confirm Gaussian distribution and, subsequently, either Brown–Forsythe ANOVA with Dunnett's multiple comparisons test for normally distributed endpoints (filtering rate, length of the reproductive period, lifetime fecundity, individual protein content and RNA:protein ratio) or Kruskal–Wallis (KW) with Dunn's multiple comparisons test for those significantly deviating from the normal distribution (age at first reproduction) were used to evaluate treatment effects. The significance level was set at 5% (*p* < 0.05).

## 3. Results

### 3.1. Lifespan

**Figure 1.**
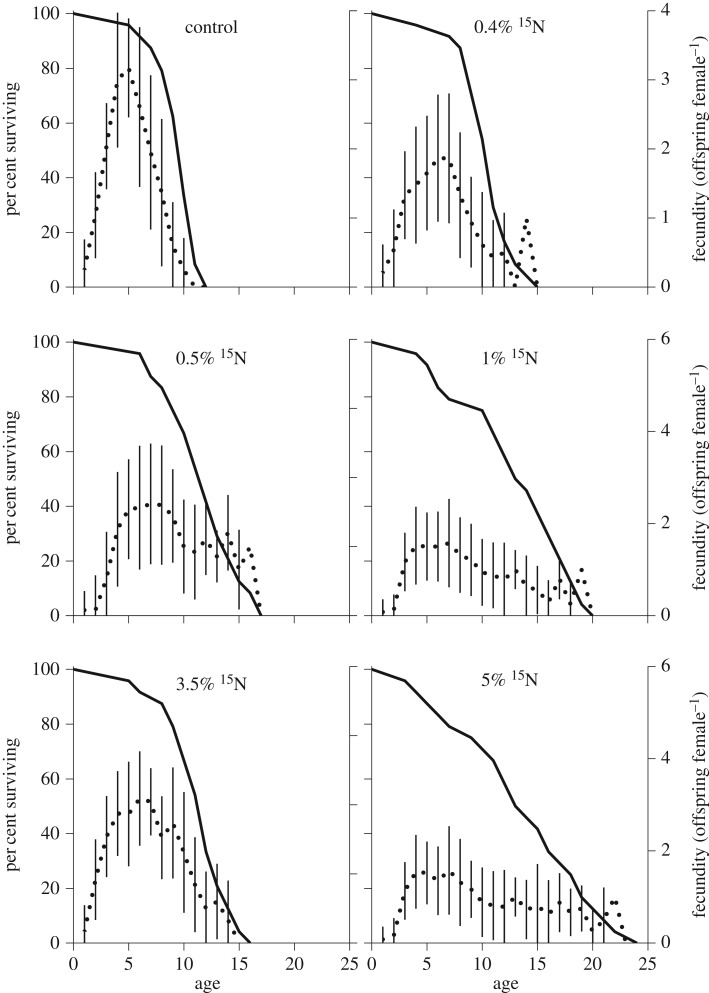
Survival (solid line) and age-specific fecundity (dotted line, mean and standard deviation) of the rotifer *Brachionus plicatilis* fed algal diets with varying ^15^N-enrichment (0.4 to 5%); non-enriched algae were used as the control diet. See electronic supplementary material, tables S1–S5 for statistical comparisons and electronic supplementary material, table S6 for primary data.

**Figure 2.**
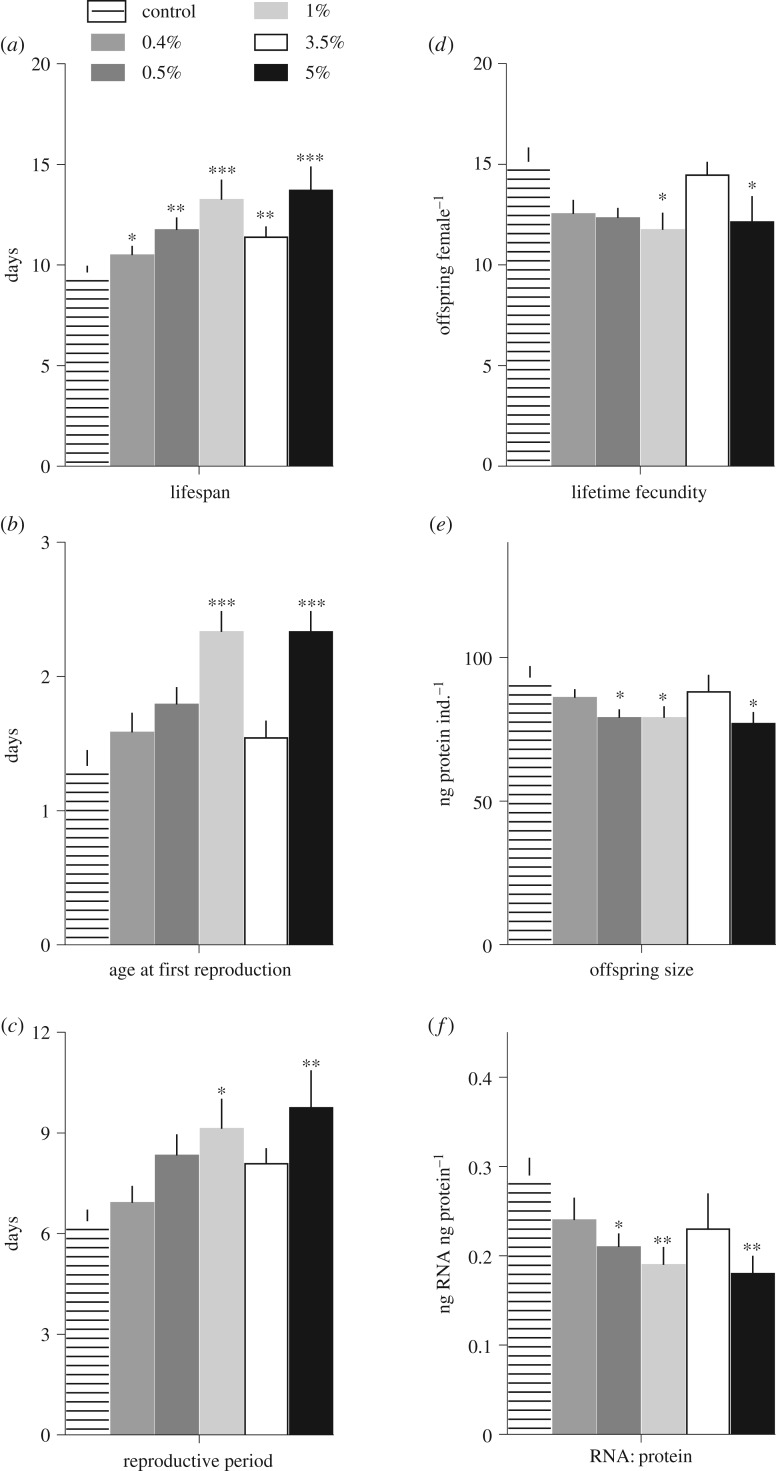
Variation in life-history traits (mean and standard deviation) of *Brachionus plicatilis* fed algal diets with varying ^15^N-enrichment (control and 0.4 to 5% ^15^N). Lifespan (*a*), age at first reproduction (*b*), duration of the reproductive period (*c*), lifetime fecundity (*d*), offspring size measured as protein content (*e*) and RNA:protein ratio in neonates (*f*); asterisks indicate significant differences from the control (*: *p* < 0.05, **: *p* < 0.01, ***: *p* < 0.001). See electronic supplementary material, tables S1–S5 for details on the statistical comparisons.

The ^15^N-enriched diets affected the shape of the survival curves (figure 1), and median lifespan significantly increased in rotifers exposed to ^15^N-enriched food compared with the control group (table 1 and figure 2). The greatest change corresponded to approximately 40% increase in the mean lifespan, with maximal longevity values differing approximately twofold between the animals receiving the 5 at% ^15^N diet and the controls (12 versus 24 d).

**Table 1.** Fold-change and log-rank (Mantel–Cox) analysis of the lifespan in *Brachionus plicatilis* exposed to diets with varying ^15^N-enrichment in comparison to the control (0.37 at% ^15^N concentration); see figures 1 and 2 for details and the electronic supplementary material, table S6, for the primary data.

treatment	fold-change	*χ*^2^	*p*-values
0.4 at%	1.09	4.069	0.044
0.5 at%	1.22	11.40	0.0007
1.0 at%	1.38	16.66	<0.0001
3.5 at%	1.18	11.40	0.0007
5.0 at%	1.42	15.92	<0.0001
log-rank test for trend		26.07	<0.0001

### 3.2. The timing of reproduction

Rotifers receiving 1 and 5 at% ^15^N-enriched diets had significantly delayed reproduction (KW statistic = 38.85, *p* < 0.0001). Moreover, the age at first reproduction increased with increasing ^15^N concentration, with the first reproduction occurring on average 1 day later than in the controls. However, the 3.5 at% ^15^N treatment deviated from this trend and was not significantly different from the control (figure 2*b*; see also the electronic supplementary material, table S1). Moreover, the reproductive period in the rotifers receiving ^15^N-enriched diets was significantly longer (ANOVA: *F*_5, 138_ = 3.189; *p* < 0.008; Brown–Forsythe test: *F*_5, 138_ = 10.79; *p* < 0.0001; figure 2*c*), and in the most affected treatment (5 at% ^15^N), it was more than 30% longer compared with the control. Again, the 3.5 at% ^15^N treatment deviated from this trend, being non-significantly different from the control (figure 2*c*; see also the electronic supplementary material, table S2).

### 3.3. Reproductive output and offspring quality

The dynamics of the egg-laying in the rotifers receiving ^15^N-enriched diets differed from the control (figure 1). As lifespan increased, both the average age-specific fecundity and lifetime fecundity decreased (Pearson *r*: *R*^2^ = 0.77, *p* < 0.02 and *R*^2^ = 0.57, *p* < 0.09, respectively). The latter differed significantly among the treatments (ANOVA: *F*_5, 138_ = 2.834; *p* < 0.02; Brown–Forsythe test: *F*_5, 138_ = 4.278; *p* < 0.002), reaching in the most affected treatments only 60–70% of the control values. A deviating response was observed in the rotifers exposed to 3.5 at% ^15^N that showed lifetime fecundity similar to that in the control (figure 2*d*; electronic supplementary material, table S3). Moreover, offspring size measured as individual protein content (ANOVA: *F*_5, 138_ = 2.528; *p* < 0.04; figure 2*e*; electronic supplementary material, table S4) and RNA:protein ratio (ANOVA: *F*_5, 138_ = 2.883; *p* < 0.02; figure 2*f*) showed a similar pattern, with significant decreases for both growth proxies in all treatments but 3.5 at% ^15^N (electronic supplementary material, table S5).

### 3.4. Feeding activity

There were no significant differences in filtering rate between the treatments (ANOVA: *F*_5,102_ = 0.11, *p* > 0.05) and no apparent trend related to ^15^N concentration in the food (electronic supplementary material, figure S1). However, the between-replicate variability was relatively high (CV% 15–17%) and power analysis indicated that this experimental design was sufficient for detecting between-treatment differences that exceed approximately 20%. The food depletion was similar between the treatments being in the range of 13–18%, and no mortality occurred during the feeding trial.

## 4. Discussion

We found significant effects of the dietary ^15^N-enrichment on the rotifer life-history traits under controlled conditions and invariant food intake. In particular, the extended lifespan and reproductive toxicity were observed in the animals receiving ^15^N-enriched food. Moreover, these changes were associated with decreased protein allocation to the offspring as well as their growth potential, thus potentially affecting the next generation. In addition, the effect of the ^15^N concentration was not monotonous as evidenced by a differential pattern of the responses in the rotifers from 3.5 at% ^15^N treatment compared with all other treatments with ^15^N-enriched diet. More specifically, the lifetime fecundity, timing of reproduction and offspring quality were similar between the rotifers fed 3.5 at% ^15^N diet and the control, albeit the lifespan in the former group was approximately 18% longer. These findings support the existence of kinetic isotope effects in the biochemical reactions occurring in multicellular consumers exposed to isotopically enriched diets. They also provide evidence for the occurrence of enhanced growth at 3.5 at% ^15^N as predicted by the isotopic resonance hypothesis [12]; notably, with no penalty for the lifespan.

Heavy isotopes incorporated into biological molecules, such as lipids and amino acids, have been proposed to confer cell resistance to reactive oxygen species (ROS) or reactive nitrogen species (RNS), metabolic products which cause chemical damage [10]. For example, replacement of the bis-allylic hydrogen atoms with deuterium atoms arrests PUFA autoxidation due to the isotope effect, rendering cells more resistant to oxidative damage [27]. According to the free-radical theory of ageing, the enhanced resistance to oxidative stress would result in an increased lifespan (e.g. [28]), as was observed in the rotifers receiving ^15^N-enriched food (figure 1). The animals receiving diets containing 0.4–5 at% ^15^N had lifespan extended up to 40% with concomitant changes in the timing of reproduction and decreased lifetime fecundity by 30–40%. The decline in the reproductive output in combination with increased longevity is typically observed in rotifers on low-calorie diets [29] and under exposure to some chemical compounds [30]. However, in this case, both food intake (electronic supplementary material, figure S1) and elemental composition of the algae (at least, %C and %N) were virtually invariable among the treatments. Therefore, the observed changes in the life histories can only be attributed to the differential metabolic processing of macromolecules with varying proportion of ^15^N, the stability of these molecules, and subsequent change in reproductive output and longevity. The elemental composition is not sufficient to comprehensively characterize food quality, and it is also possible that nutritional value of the algae grown in the ^15^N-enriched media was lower compared with the non-enriched algae, which resulted in the compromised diet. Recently, profound changes in growth dynamics of green algae grown at similar levels of ^15^N-enrichment (0.5–5 at%) were demonstrated, with the overall response being indicative of stress [7]. Therefore, it is probable that biochemical composition of the algae exposed to ^15^N-labelling was also affected resulting in suboptimal nutritional quality for the rotifers.

In addition to increased stability, the isotope labelling of yeast has been reported to affect the molecular architecture of proteins [31], raising questions about possible differences in functionality of the isotope-labelled proteins. In bacteria grown in ^15^N-enriched medium, multiple effects on the key biosynthetic and metabolic pathways, such as citric acid cycle (a metabolic pathway that unifies carbohydrate, fat and protein metabolism), pyruvate production and glycolysis were observed in concert with growth inhibition [5]. As these pathways are highly evolutionarily conserved, it is not particularly surprising to observe alterations in energy allocation and growth of the rotifers fed a ^15^N-enriched food. The observed increase in the age at first reproduction (figure 2*b*) and the lower protein content of the neonates (figure 2*e*) can be related to a decrease in somatic growth or energy allocation to reproduction. A dual-labelling of the algae, e.g. with ^13^C and ^15^N as commonly used in physiological and ecological studies, would probably introduce even more complex interactions as suggested by differential growth responses observed in fungi grown on ^15^N-labelled, double ^15^N- and ^13^C-labelled, and unlabelled media [8].

The effects of heavy isotopes on organism metabolic responses and life histories would be negligible over short periods but could become apparent after accumulation resulting from massive flux, such as in studies employing isotope enrichment. Also, both positive and negative effects on biosynthesis, as well as complex life-history trade-offs, can be expected, depending on the isotope in question, enrichment level and its proximity to the resonance concentration as well as biology of the test species. In fish fed a series of diets with a range of δ^13^C (−22.9 to −6.6‰) and δ^15^N (6.5 to 1586‰) and invariant C : N ratio, no treatment-related differences in fish growth were observed over several weeks of the experiment. However, the fish C : N ratio varied among the treatments, with highest values occurring in the diets with intermediate (0.4–0.7 at% ^15^N and 1.09 at% ^13^C) concentrations of the heavy isotopes [32]. This non-monotonous response indicates possible effects of the isotope enrichment, the element and the combination of the two. In bacteria, a significant increase in the maximum growth rate occurred at certain isotope concentrations (0.37, 3.5 and 9.7 at% for 15 N; [6], and the effect was consistent with the resonance conditions predicted by the isotopic resonance theory [12]. The reproductive endpoints of the rotifers in the 3.5 at% ^15^N treatment (i.e. the resonance concentration) were similar to those in the controls, whereas the offspring produced in other ^15^N treatments were fewer and of lower quality. Thus, the rotifers receiving ^15^N resonance diet had higher fitness, with a longer lifespan and similar or greater lifetime fecundity, compared with all other treatments, including control. The mechanisms of this phenomenon are far from being understood, and it will be important in future studies to examine the molecular and physiological basis of these responses.

Ecologists have now switched from application of radioactive tracers to stable isotopes, due to the high availability of a large variety of compounds and analytical facilities, lower cost and lower health risks during handling. However, unlike the radioisotope techniques, stable isotope labelling often employs very high concentrations of heavy isotope to ensure detectability and facilitate the use of small samples for isotope analysis. For example, substrate ^15^N can reach as high as 50 at% [13–15,33,34]. Particularly relevant are the studies aimed at quantifying isotopic turnover rate in animal tissue because they often involve diet-shift experiments with isotope-labelled diets [35]. Needless to say that turnover values estimated for ^13^C and ^15^N in such experiments are likely to be underestimated, should the kinetic effect occur.

Isotope labelling should not be applied without understanding implications of KIEs in our study systems. These findings demonstrate for the first time that ^15^N-labelling may affect any life-history trait of the multicellular consumer and underline the importance of accounting for such effects when using stable isotope labelling in experimental studies. Although the popularity of isotope labelling experiments is growing, there has been no systematic evaluation of KIE in various biological systems. Despite calls for more experimental studies in animal isotopic ecology, field studies still outnumber laboratory studies [36]. Also, mechanistic models of isotopic incorporation rates are scarce [37]. To incorporate KIEs in these models, controlled experiments using combined approaches, including physiology, metabolomics and life histories, are required. Future research into how isotope-enrichment interacts with physiological rates and traits might yield new insights into the mechanisms underpinning animal development and ecology, with implications for physiology of health and disease [38], longevity [9,11], metabolomics [39] and astrobiology [12].

## Supplementary Material

Tables S1-S5. Multiple comparison tests for life history traits.

## Supplementary Material

Table S6. Life table data for Brachiomus plicatilis exposed to 15N-enriched algal food.

## Supplementary Material

Figure S1. Food intake of Brachionus plicatilis exposed to 15N-enriched algal food.

## Data accessibility

All data are provided as an Excel table in the electronic supplementary material, table S6.
