# Supplementary material for: Shifts in rotifer life history in response to stable isotope enrichment: testing theories of isotope effects on organismal growth
Source: R Soc Open Sci. 2017 Mar 29;4(3):160810. doi: 10.1098/rsos.160810 (PMC5383824; doi:10.1098/rsos.160810)
Supplement: Tables S1-S5. Multiple comparison tests for life history traits. [file rsos160810supp1.pdf]

Shifts in rotifer life history in response to stable isotope enrichment: testing theories of isotope effects on organismal growth

Elena Gorokhova

Electronic Supplementary Materials

Appendix S1, Tables S1-S5.

Table S1. Dunn's multiple comparison tests for age at first reproduction in *Brachionus plicatilis* receiving diets with varying ^{15}N enrichment. All treatments are compared to the control (0.37 at% ^{15}N concentration); ns: not significant, ****: p < 0.0001. See also Figure 2B.

Pairwise comparison	Mean rank diff.	Summary
Control vs. 0.4 at%	-13.92	ns
Control vs. 0.5 at%	-23.98	ns
Control vs. 1.0 at%	-52.17	****
Control vs. 3.5 at%	-10.77	ns
Control vs. 5.0 at%	-52.17	****

Table S2. Dunnett's multiple comparison tests for the duration of the reproductive period *Brachionus plicatilis* receiving algal diets with varying ^{15}N enrichment. All treatments are compared to the control (0.37 at% ^{15}N concentration); ns: not significant, *: p < 0.05; ** p < 01. See also Figure 2C.

Pairwise comparison	Mean diff.	95%-CI of diff.	Summary
Control vs. 0.4%	-0.5833	-3.147 to 1.980	ns
Control vs. 0.5%	-1.958	-4.522 to 0.6050	ns
Control vs. 1%	-2.750	-5.313 to -0.1866	*
Control vs. 3.5%	-1.708	-4.272 to 0.8550	ns
Control vs. 5%	-3.375	-5.938 to -0.8116	**

Table S3. Dunnett's multiple comparison tests for the lifetime fecundity in *Brachionus plicatilis* receiving algal diets with varying ^{15}N enrichment. All treatments are compared to the control (0.37 at% ^{15}N concentration); ns: not significant, *: $p < 0.05$; ** $p < 01$. See also Figure 2D.

Pairwise comparison	Mean diff.	95%-CI of diff.	Summary
Control vs. 0.4%	2.583	-0.3765 to 5.543	ns
Control vs. 0.5%	2.792	-0.1682 to 5.751	ns
Control vs. 1%	3.375	0.4152 to 6.335	*
Control vs. 3.5%	0.6667	-2.293 to 3.626	ns
Control vs. 5%	3.000	0.04017 to 5.960	*

Table S4. Dunnett's multiple comparison tests for the individual protein content of the *Brachionus plicatilis* neonates produced by mothers receiving diets with varying ^{15}N enrichment. All treatments are compared to the control (0.37 at% ^{15}N concentration); ns: not significant, *: $p < 0.05$; ** $p < 01$. See also Figure 2E.

Pairwise comparison	Mean diff.	95%-CI of diff.	Summary
Control vs. 0.4%	7.000	-6.508 to 20.51	ns
Control vs. 0.5%	14.00	0.6497 to 27.35	*
Control vs. 1%	14.00	0.3200 to 27.68	*
Control vs. 3.5%	5.000	-9.548 to 19.55	ns
Control vs. 5%	16.00	0.5104 to 31.49	*

Table S5. Dunnett's multiple comparison tests for the RNA:protein ratio in the neonates of *Brachionus plicatilis* produced by mothers receiving diets with varying ^{15}N enrichment. All treatments are compared to the control (0.37 at% ^{15}N concentration); ns: not significant, *: p < 0.05; ** p < 01. See also Figure 2F.

Pairwise comparison	Mean diff.	95%-CI of diff.	Summary
Control vs. 0.4%	0.0500	-0.03029 to 0.1303	ns
Control vs. 0.5%	0.0800	0.0006538 to 0.1593	*
Control vs. 1%	0.1000	0.01869 to 0.1813	**
Control vs. 3.5%	0.06000	-0.02647 to 0.1465	ns
Control vs. 5%	0.1100	0.01794 to 0.2021	*
