# Supplementary material for: Shifts in rotifer life history in response to stable isotope enrichment: testing theories of isotope effects on organismal growth
Source: R Soc Open Sci. 2017 Mar 29;4(3):160810. doi: 10.1098/rsos.160810 (PMC5383824; doi:10.1098/rsos.160810)

Shifts in rotifer life history in response to stable isotope enrichment: testing theories of isotope effects on organismal growth

Elena Gorokhova

Electronic Supplementary Materials

Appendix S2

Figure S1. Food intake of *Brachionus plicatilis* offered algae with varying ^{15}N enrichment (control and 0.4 to 5 at% ^{15}N).

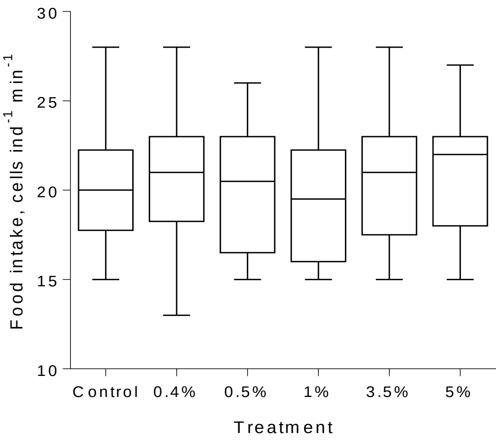

Supplement: Figure S1. Food intake of Brachionus plicatilis exposed to 15N-enriched algal food. [file rsos160810supp3.pdf]
